# The biological significance of brain barrier mechanisms: help or hindrance in drug delivery to the central nervous system?

**DOI:** 10.12688/f1000research.7378.1

**Published:** 2016-03-10

**Authors:** Norman R. Saunders, Mark D. Habgood, Kjeld Møllgård, Katarzyna M. Dziegielewska

**Affiliations:** 1 Department of Pharmacology & Therapeutics, University of Melbourne, Parkville, Victoria, Australia; 2 Institute of Cellular and Molecular Medicine, University of Copenhagen, Copenhagen, Denmark

**Keywords:** Blood-brain barrier, cerebrospinal barrier, CSF-brain barrier, transporters, tight junctions, drug delivery

## Abstract

Barrier mechanisms in the brain are important for its normal functioning and development. Stability of the brain’s internal environment, particularly with respect to its ionic composition, is a prerequisite for the fundamental basis of its function, namely transmission of nerve impulses. In addition, the appropriate and controlled supply of a wide range of nutrients such as glucose, amino acids, monocarboxylates, and vitamins is also essential for normal development and function. These are all cellular functions across the interfaces that separate the brain from the rest of the internal environment of the body. An essential morphological component of all but one of the barriers is the presence of specialized intercellular tight junctions between the cells comprising the interface: endothelial cells in the blood-brain barrier itself, cells of the arachnoid membrane, choroid plexus epithelial cells, and tanycytes (specialized glial cells) in the circumventricular organs. In the ependyma lining the cerebral ventricles in the adult brain, the cells are joined by gap junctions, which are not restrictive for intercellular movement of molecules. But in the developing brain, the forerunners of these cells form the neuroepithelium, which restricts exchange of all but the smallest molecules between cerebrospinal fluid and brain interstitial fluid because of the presence of strap junctions between the cells. The intercellular junctions in all these interfaces are the physical basis for their barrier properties. In the blood-brain barrier proper, this is combined with a paucity of vesicular transport that is a characteristic of other vascular beds. Without such a diffusional restrain, the cellular transport mechanisms in the barrier interfaces would be ineffective. Superimposed on these physical structures are physiological mechanisms as the cells of the interfaces contain various metabolic transporters and efflux pumps, often ATP-binding cassette (ABC) transporters, that provide an important component of the barrier functions by either preventing entry of or expelling numerous molecules including toxins, drugs, and other xenobiotics.

In this review, we summarize these influx and efflux mechanisms in normal developing and adult brain, as well as indicating their likely involvement in a wide range of neuropathologies.

There have been extensive attempts to overcome the barrier mechanisms that prevent the entry of many drugs of therapeutic potential into the brain. We outline those that have been tried and discuss why they may so far have been largely unsuccessful. Currently, a promising approach appears to be focal, reversible disruption of the blood-brain barrier using focused ultrasound, but more work is required to evaluate the method before it can be tried in patients. Overall, our view is that much more fundamental knowledge of barrier mechanisms and development of new experimental methods will be required before drug targeting to the brain is likely to be a successful endeavor. In addition, such studies, if applied to brain pathologies such as stroke, trauma, or multiple sclerosis, will aid in defining the contribution of brain barrier pathology to these conditions, either causative or secondary.

## Introduction

The term blood-brain barrier has a long history. Its current usage describes the structural, physiological, and molecular mechanisms that control the exchange (entry and exit) of molecules between the blood and the brain. The sum of these mechanisms results in the characteristically stable internal environment of the brain, both during development and in the adult. This has been an often confused and misunderstood field of neuroscience.

The main aim of this review is to explain what is known about brain barrier mechanisms and why understanding these mechanisms is fundamental to understanding normal brain development and normal brain function and how disorders of brain barrier mechanisms may contribute to a range of neuropathological conditions. We suggest that the neuroscience community should pay more attention to this topic and we advocate the need for new researchers to move into this intriguing and important field, as major advances in many fields have often come from an influx of new people unfettered by the prevailing dogmas.

The other focus of this review will be to consider the clinically important problem of developing ways to deliver drugs to the brain for treating neurological and psychiatric disorders. This has been a major effort in the blood-brain barrier field for the past 20–30 years but has yielded little of practical value. We list the diverse attempts that have been tried, we analyze some of the possible reasons why they have been unsuccessful, and we suggest some alternative/new approaches to the problem.

## What is meant by the term “blood-brain barrier”?

The use of the term “barrier” is in many ways unfortunate ^1^, as for those outside the field it disguises the multiplicity of mechanisms involved. Perhaps this also explains the almost exclusive focus of people interested in pathological conditions involving the blood-brain barrier on tests of its integrity, largely ignoring until recently the numerous cellular mechanisms at the various blood-brain interfaces that may be disrupted. Almost all of the early work on the blood-brain barrier involved the use of dyes, which could be visualized. This field has recently been reviewed with translations from key oft-cited papers published in their original languages showing that many of the citations were incorrect ^2^. To set the record straight, it was Lena Stern who was the first to coin the term blood-brain barrier (“barrière hémato-encéphalique” ^3^) and not, as often cited, Ehrlich ^4^, Lewandowsky ^5^, or Goldmann ^6^. The current understanding of the term “blood-brain barrier” is that it covers a number of morphological entities and a plethora of cellular transport mechanisms both inward and outward, which we next describe briefly.

## Morphology of blood-brain barrier interfaces

**Figure 1.**
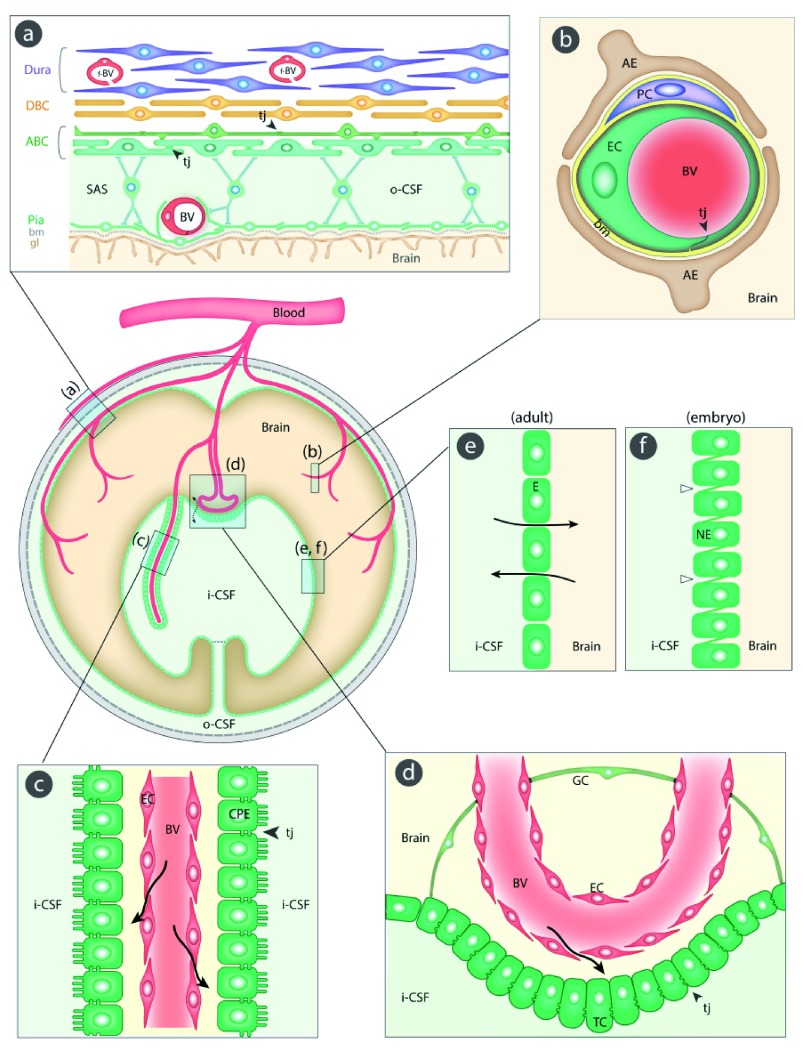
Schematic diagram (center left) of the five main barrier interfaces (a–e) in the brain and an additional one in the embryo (f). The barrier-forming cellular layers at each interface are colored green. ( **a**) *The meningeal barrier* is structurally the most complex of all the brain barriers. Barrier-forming cells are the outer layer of the arachnoid membrane (the arachnoid barrier cells [ABC]); these have tight junctions (arrowheads) between adjacent cells forming a barrier between the outer cerebrospinal fluid (o-CSF) in the subarachnoid space (SAS) and more superficial dural layers (dural border cells [DBC] and the dura mater). Blood vessels (BV) in the SAS have tight junctions with similar barrier characteristics as cerebral blood vessels without surrounding pericytes and astrocytic end-feet ^11– 13^. Blood vessels within the dura mater are fenestrated (f-BV); bm = basement membrane, gl = glia limitans. ( **b**) *The blood-brain barrier* is situated at the level of cerebral blood vessels (BV). Tight junctions (tj, arrowhead) are present between the endothelial cells (EC) restricting the paracellular cleft ( 11 and Text Box); bm = basement membrane, PC = pericytes, AE = end feet from astroglial cells. ( **c**) *The blood-CSF barrier* is situated in the choroid plexus within each brain ventricle. Barrier-forming cells are the epithelial cells (CPE), which have tight junctions ^11^ at their apical side (CSF facing, arrowheads). Blood vessels (BV) are fenestrated and do not form a barrier (arrows); apical microvilli increase exchange surface of epithelial cells to the internal CSF (i-CSF). ( **d**) Circumventricular organs (including median eminence, pineal gland, area postrema, subfornical organ). Blood vessels have permeability characteristics similar to elsewhere in the body and have the functional property of allowing feedback penetration of peptide hormones controlled by the hypothalamic-pituitary axis. These peptides and other molecules are prevented from entering the CSF by tanycytes (TC), the specialized ependymal cells of these brain areas, connected by tight junctions between their apices (arrowhead); entry into the rest of the brain is prevented by tight junctions between astroglial cells (GC ^19, 20^). Away from the tanycyte layer, ependymal cells lining the ventricular system are linked by gap junctions that do not hinder free exchange between the CSF and brain interstitial fluid (broken arrow). ( **e**) *Ependyma in adult brain*. Apart from areas where there are specialized tanycytes, ependymal cells are linked by gap junctions that do not restrict exchange of even large molecules, such as proteins, between CSF and interstitial space of brain (solid arrows). ( **f**) *The embryonic CSF-brain barrier.* In early brain development, strap junctions (open arrowheads) are present between adjacent neuroepithelial cells (NE); these form a barrier restricting the movement of larger molecules, such as proteins, but not smaller molecules.

There are six interfaces to be considered. Figure 1 illustrates their sites and main morphological features. An essential component of all interfaces with barrier properties is the presence of specialized junctions between the cells of the interface. In most of the barriers, these junctions are tight junctions; they restrict the movement of molecules between the endothelial and the epithelial cells. As a direct consequence of this restriction, the intercellular junctions have the important functional effect of allowing the numerous transporters within individual cells to operate over the large surface of the barrier interfaces; without this permeability restriction, the inward and outward transporter mechanisms would be ineffective. In recent years, it has become increasingly apparent that there is a much greater complexity involved in the structural organization of the brain barriers; in the case of the blood-brain barrier itself, this includes astrocytes, pericytes, basement membrane, and extracellular matrix ( Figure 1). However, there is much to be learned about the precise role of individual morphological components of the brain barriers and their interactions in normal and pathological brains ^7– 10^. We shall now consider each barrier interface in turn: (a) *the meningeal barrier*, shown in Figure 1(a), is structurally the most complex of all the brain barriers and is situated at the meninges (pia, arachnoid, and dura mater). The barrier-forming cells are the outer layer of the arachnoid membrane (the arachnoid barrier cells), which have tight junctions between adjacent cells forming a physical barrier between the outer cerebrospinal fluid (CSF) in the subarachnoid space and more superficial dural layers (dural border cells and the dura mater). The blood vessels in the subarachnoid space have tight junctions with similar barrier characteristics as cerebral blood vessels, although lacking the surrounding pericytes and astrocytic end-feet ^11– 13^. In contrast, blood vessels within the dura mater are fenestrated; other important components of the barrier are the basement membrane and glia limitans. (b) *The blood-brain barrier*, shown in Figure 1(b), is situated at the level of cerebral blood vessels between the lumen of the vessel and brain parenchyma. Tight junctions are present between the endothelial cells restricting permeability of the paracellular cleft ( 11 and Text Box). A basement membrane and extracellular matrix ^14^ surround both the endothelial cells and the pericytes ^15, 16^. End feet from astroglial cells progressively encircle cerebral blood vessels during development ^17^. These cellular structures are known collectively as the neurovascular unit ^18^. (c) *The blood-CSF barrier*, shown in Figure 1(c), is situated in the choroid plexus within each brain ventricle. In contrast to other cerebral blood vessels, the endothelial cells forming choroid plexus blood vessels are fenestrated and do not form a barrier. The barrier-forming cells are the epithelial cells, which have tight junctions ^11^ at their apical (CSF) side. Choroid plexus cells have microvilli on their apical side, increasing their exchange surface to the internal CSF. (d) *Circumventricular organs*, shown in Figure 1(d). These include the median eminence, pineal gland, area postrema, and subfornical organ. The blood vessels have permeability characteristics similar to elsewhere in the body and have the functional property of allowing feedback penetration of peptide hormones controlled by the hypothalamic-pituitary axis. These peptides and other molecules are prevented from entering the CSF by tanycytes, the specialized ependymal cells of these brain areas, connected by tight junctions between their apices; entry into the rest of the brain is prevented by tight junctions between astroglial cells ^19, 20^. (e) *Ependyma* in adult brain, shown in Figure 1(e). Apart from areas where there are specialized tanycytes, ependymal cells are linked by gap junctions that do not restrict exchange of even large molecules, such as proteins, between CSF and interstitial space of brain ^11, 21^. (f) *The embryonic CSF-brain barrier*, shown in Figure 1(f). In the ventricular zone is a temporary barrier between the CSF and brain parenchyma ^21^. In early brain development, strap junctions are present between adjacent neuroepithelial cells; these form a physical barrier restricting the movement of larger molecules, such as proteins, but not smaller molecules ^22, 23^. At later stages of development and in the adult brain, these strap junctions are no longer present when this interface becomes ependyma.

## Transport mechanisms at barrier interfaces

### Control of the interstitial ionic environment of the brain

**Figure 2.**
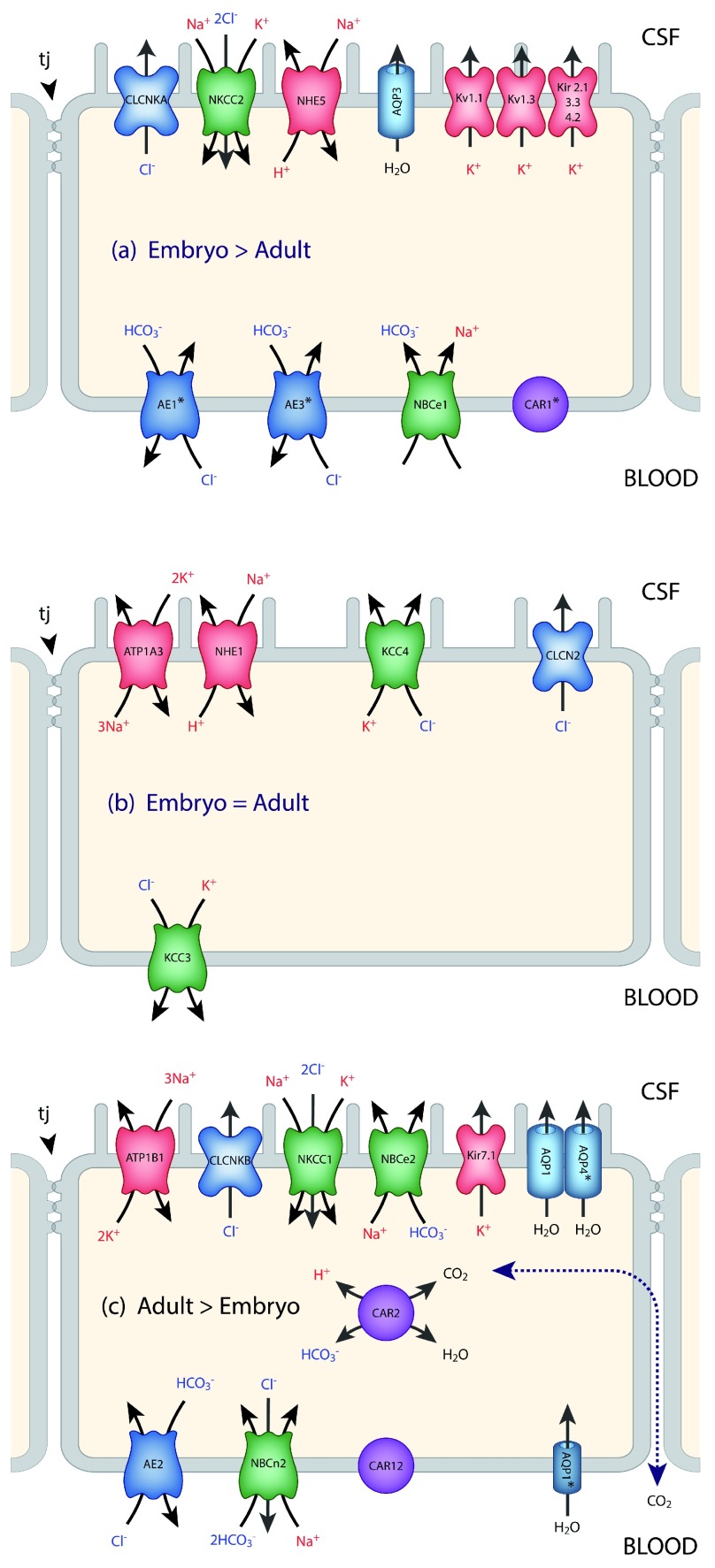
Transporters and ion channels in choroid plexus epithelial cells. Data for the localization of transporters and ion channels are from Damkier *et al.* ^25^ and Brown *et al.* ^96^. CSF secretion results from coordinated transport of ions and water from basolateral membrane to cytoplasm, then sequentially across apical membrane into ventricles ^25^. The genes for many of these transporters and ion channels are differentially expressed in the embryo compared to the adult. This is represented in the three panels. It is emphasized that this represents differential expression, not the absence of a gene at one age (details are in 35). On the plasma-facing membrane is parallel Cl ^-^/HCO _3_^-^ exchange (AE2 [ *Slc4a2*] > in adult and AEI [ *Slc4a1*], AE3 [ *Slc4a3*] > in embryo) and Na ^+^/HCO _3_^-^ co-transport (NBC1 [ *Slc4a4*] > in embryo) with net function bringing Cl ^-^ into cells in exchange for HCO _3_^-^^97^. Also basolaterally located is an Na-dependent Cl ^-^/HCO _3_^-^ exchange (NCBE [ *Slc4a10*] > in adult) that modulates pH and perhaps CSF formation ^98^. Apical Na ^+^ efflux by NHE5 ( *Slc9a5* > in embryo) and ATB1 ( *Atb1b1* [Na ^+^/K ^+^-ATPase] > in adult) maintains a low cell Na ^+^ that sets up a favorable basolateral gradient to drive Na ^+^ uptake ^99^. Na ^+^ is extruded into CSF mainly via the Na ^+^/K ^+^-ATPase pump (ATB1 [ *Atb1b1*]) and, under some conditions, the Na ^+^/K ^+^-Cl ^-^ co-transporter NKCC1, *Slc12a2* (see 100 for review). Aquaporin (AQP1/3/4) channels on CSF-facing membrane mediate water flux into ventricles ^101^. Polarized distribution of carbonic anhydrase (CAR) and Na ^+^/K ^+^-ATPases, and aquaporins, enable net ion and water translocation to CSF (see 100 and 102 for reviews). CLCKA (CLCK1) is an inwardly rectifying chloride channel; its gene ( *Clcnka*) in embryonic choroid plexus is expressed many orders of magnitude higher than in the adult. *Clcnkb* is expressed at a higher level in the adult. CAR2 has an intracellular distribution and is functionally important for catalyzing the equilibrium that generates H ^+^ and HCO _3_^-^, which is an important part of the mechanism secreting CSF. There are many more channels that show age-related differential expression in choroid plexus, the functions of which are unclear ^26^.

**Figure 3.**
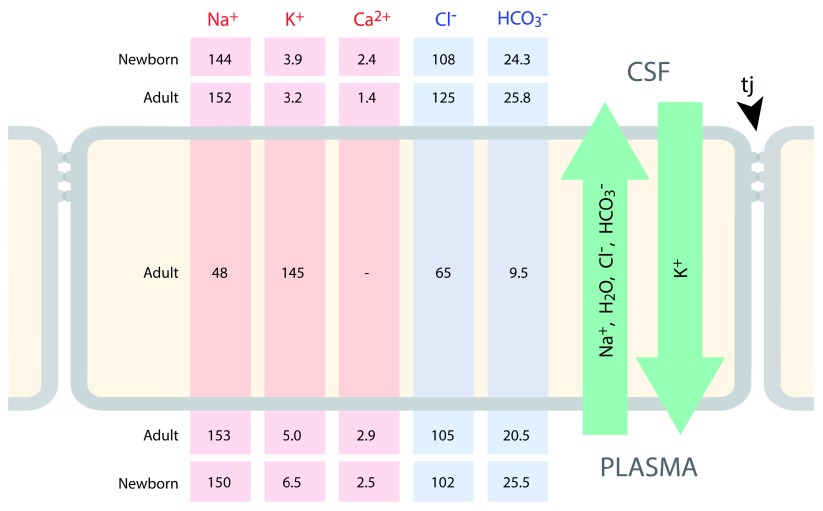
Ion gradients between CSF and plasma in developing and adult rat brain. A characteristic of CSF is its stable ionic composition that differs from that of plasma to an extent that cannot be explained by ultrafiltration, as was once thought ^24^. Data for CSF and plasma (m-equiv/L water) are from 103 and for intracellular ions (mmol/L water) from Figure 8 in 104. The gradients are the consequence of the complex interactions between enzymes (notably carbonic anhydrase) ion transporters and ion channels, as illustrated in Figure 2. The CSF secretion rate in the embryo and newborn is much lower than in the adult ^105– 107^, which is perhaps explained by the much lower expression of carbonic anhydrase and ATPases in the developing choroid plexus.

This is absolutely critical for normal function of the brain. As Hugh Davson once put it (paraphrased), without this control our sensory experience would be limited to a series of flashes and bangs. The ionic composition of the interstitial fluid is usually taken to be synonymous with the composition of CSF ^24^. This, plus the obvious practical point that it is relatively easy to sample CSF, has led to a focus on CSF and the choroid plexus in both the adult ^25^ and the developing ^26^ brain; however, CSF composition generally does not reflect blood-brain barrier function (see Text Box). There is a good correlation between expression levels of transporters and ionic concentrations at different ages, at least in the rat ( Figure 2 and Figure 3).

### Influx mechanisms

**Figure 4.**
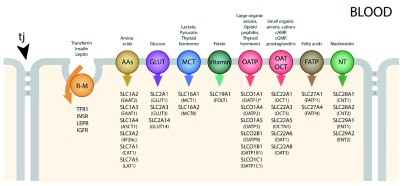
Summary of inward transporter mechanisms in cerebral endothelial cells. Individual transporters shown are ones identified in human material ^29^. Tight junctions (tj) between adjacent cells prevent the paracellular passage of hydrophilic compounds. R-M = receptor-mediated, GLUT = glucose transporters, NTs = nucleoside transporters, AAs = amino acid transporters (includes LAT), OATP = organic anion transporting polypeptides, OAT = organic anion transporters, OCT = organic cation transporters, MCT = monocarboxylate transporters, FATP = fatty acid transport protein. Many of these transporters are solute linked carriers (SLCs). Both SLC designations and the original abbreviations are included here. SLC1A2/EAAT2, SLC1A3/EAAT1 high-affinity glutamate, SLC1A4/ASCT1 glutamate/neutral amino acids, SLC2A1/GLUT1, SLC2A3,14/GLUT3 glucose, SLC3A2/4F2hc amino acid transporter heavy chain, SLC6A12/BGT1 neurotransmitter, SLC7A1/CAT1 cationic amino acid, y+ system, SLC7A5/LAT1 amino acid light chain, L system, SLC10A1/NTCP sodium/bile acid cotransporter. SLC16A1/MCT1, SLC16A2/MCT8 monocarboxylates, SLC19A1/RFC folate, SLC22A1/OCT1 organic cations, SLC22A3/OCTN3 organic cations, SLC22A5/OCTN2 organic cation/carnitine, SLC27A1/FATP1 fatty acid, SLC29A1/ENT1 equilibrative nucleosides, SLCO2B1/OATP2B1 organic anions, SLCO1B1/OATP1B1 organic anions. Examples of receptor-mediated transporters are insulin receptor (INSR), transferrin receptor (TFR1), leptin receptor (LEPR), low-density lipoprotein receptor (LDLR), and insulin-like growth factor receptor (IGFR).

These have been extensively studied only at the blood-brain barrier itself and at the blood-CSF barrier (choroid plexuses). A limitation of early studies with radiolabeled molecules, such as glucose and amino acids, was that it was difficult to distinguish between brain entry and incorporation of labeled molecules into metabolic pathways. This problem was solved by Oldendorf ^27^ with his short pass technique. In general, essential amino acids were transported into the brain to a greater extent than non-essential amino acids; there was also a high uptake of D-glucose ^27^. The molecular basis for these inward transport mechanisms has been extensively investigated using gene expression techniques to study the blood-brain barrier itself ^28– 30^ and also the choroid plexuses ^26, 31– 33^. These studies have revealed a plethora of genes, particularly those classed as solute linked carriers (SLCs) ^34^. These are summarized in Figure 4 for transporters identified in both the transcriptome and the proteome in human endothelial cells ^29^. Some of these carriers will transport only compounds that closely resemble endogenous substrates, as they exhibit high substrate specificity (e.g. GLUT1). Many others (e.g. organic anion transporters [OATs], OAT polypeptides [OATPs], and large amino acid transporter [LAT1]) will accept a broader range of substrates; they provide a potential route of entry into the central nervous system (CNS) for exogenous compounds. Members of the OAT family of solute carriers (SLC) are known to transport a wide range of drugs, such as aspirin, ibuprofen, and various antibiotics, and pesticides (e.g. 2,4-D-dichlorophenoxyacetic acid [2,4-D]). The plant-derived neurotoxin β- *N*-methylamino-L-alanine (MeAA) and the drug L-DOPA both have amino acid structures that allow entry via the amino acid transporter LAT1. Some environmental toxins are also able to gain entry into the CNS by attaching themselves to an endogenous substrate to be co-transported; for example, methyl-mercury (MeHg) and lead (Pb ^2+^) attached to cysteine enter via amino acid transporters specific for this amino acid, e.g. SLC1A5 and SLC7A10.

Many more *Slc* genes have been identified in the transcriptome of mouse endothelial cells ^28^. A large number was also identified in mouse lateral ventricular choroid plexuses ^33^. For comparison between the two interfaces, see 34. It is striking that the expression of some *Slc* genes in brain barriers is much higher in the developing brain ^28, 35^; this correlates with limited information of greater transport of some labeled amino acids and glucose into the developing brain, suggesting that the high expression levels correlate with transporter function (reviewed in 36). Probably several Slcs are responsible for the transport of the same molecules, indicating a significant degree of redundancy.

### Efflux mechanisms

**Figure 5.**
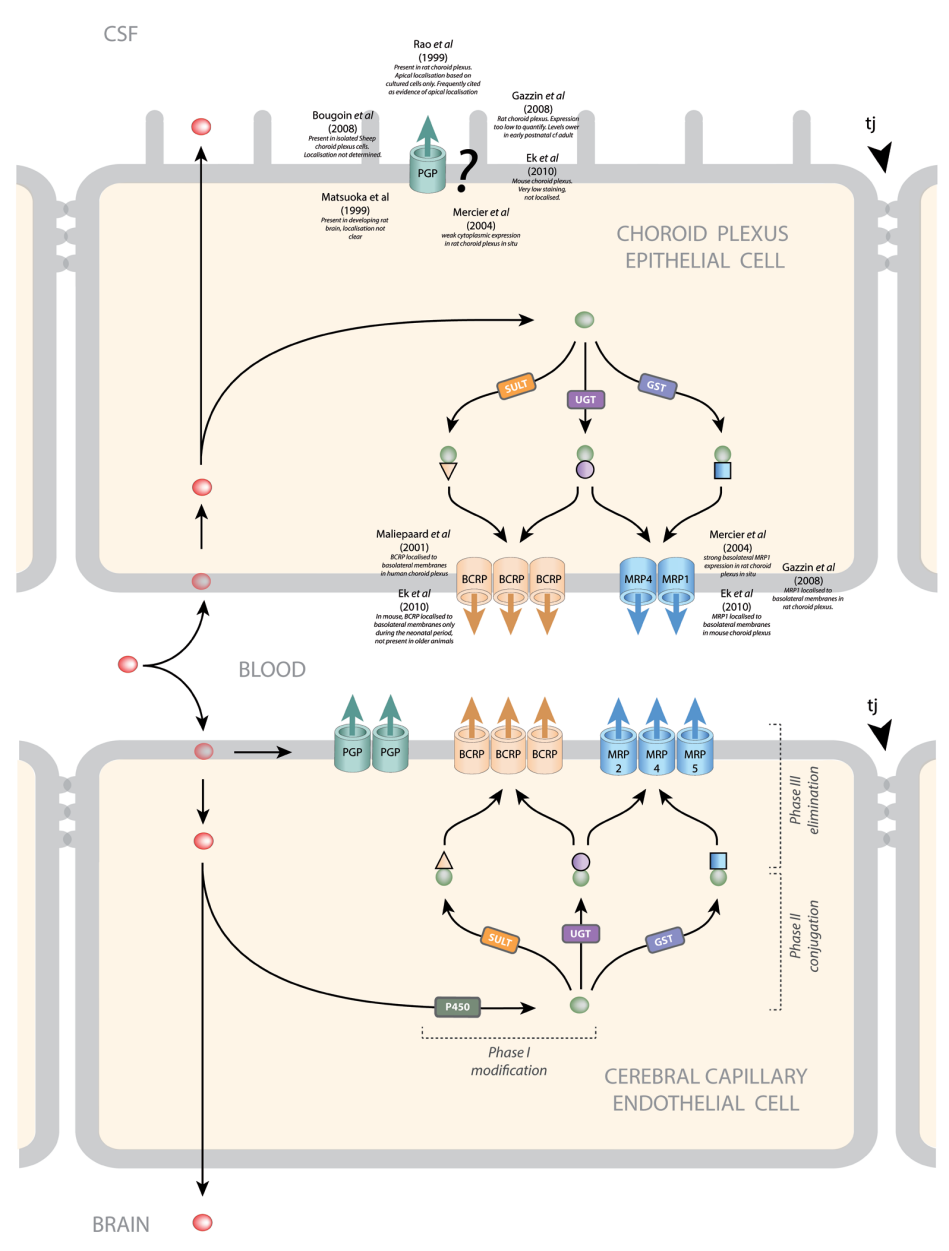
Efflux pathways in barrier-forming cells. The main efflux transporters at the blood-brain and blood-CSF interfaces are P-glycoprotein (PGP, MDR1, ABCB1), breast cancer resistance protein (BCRP, ABCG2), and several members of the multidrug resistance protein subfamily (MRP1 ABCC1, MRP2 ABCC2, MRP4 ABCC4, MRP5 ABCC5). In cerebral capillary endothelial cells (blood-brain barrier), PGP ^41– 43^, BCRP ^44, 45^, MRP2 ^46^, MRP4 ^46^, and MRP5 ^47^ are localized to the luminal membrane where they export compounds into the blood. In choroid plexus epithelial cells (blood-CSF barrier), MRP1, MRP4, and BCRP are localized to the basolateral membranes where they export compounds into the stroma of the plexus ^40, 48, 49^. The subcellular localization of PGP in choroid plexus is not clear. Some studies report staining too low to be able to determine localization ^40, 48^ or positive staining, but localization was not able to be determined ^50, 51^. Other studies report cytosolic ^52^ or subapical localization ^42^. One study has reported apical membrane localization in cultured choroid plexus epithelial cells ^53^. A common feature of these outwardly directed efflux transporters is a broad substrate specificity and considerable overlap between transporters (see 54). PGP is unusual in that it intercepts lipid-soluble compounds (red symbols) as they pass through the internal leaflet of the plasma membrane and returns them to the extracellular fluid ^55^, whereas BCRP and the MRPs bind their substrates from within the cell cytoplasm. Compounds can be exported from the cell by one or more of these efflux pathways. For example, a lipid-soluble compound that manages to avoid interception by PGP as it passes into the cell may then be metabolized by phase I enzymes (e.g. cytochrome P450 oxidases), conjugated by phase II enzymes (sulfotransferases [SULTs], uridine-diphospho-glucuronosyltransferase [UGT], or glutathione S-transferase [GST]), and exported by BCRP and/or MRP.

Of particular importance in relation to drug entry into the brain, or rather the failure of most drugs to enter the brain, are the ABC efflux transporters ^37, 38^. There are 49 members of the ABC protein superfamily ( http://nutrigene.4t.com/humanabc.htm). Many of these are efflux transporters. At the blood-brain barrier interface ( Figure 5), the efflux transporters that have been shown to be expressed and present and appear to be of particular functional importance are ABCB1 (also known as P-glycoprotein [PGP] or MDR1) and ABCG2 (breast cancer resistance protein [BCRP]). ABCC2 (multidrug resistance protein 2 [MRP2]) and ABCC4 (MRP4) have also been demonstrated at this interface ^39^. At the blood-CSF interface ( Figure 5), ABCC1 (multidrug resistance protein 1 [MRP1]) appears to be the predominant efflux transporter, but ABCC4 (MRP4) and ABCG2 (BCRP) have also been shown to be present ^39, 40^. In cerebral capillary endothelial cells (blood-brain barrier), PGP ^41– 43^, BCRP ^44, 45^, MRP2 ^46^, MRP4 ^46^, and MRP5 ^47^ are localized to the luminal membrane, where they export compounds into the blood. In choroid plexus epithelial cells (blood-CSF barrier), MRP1, MRP4, and BCRP are localized to the basolateral membranes where they export compounds into the stroma of the plexus ^40, 48, 49^. The subcellular localization of PGP in choroid plexus is not clear. Some studies report staining too low to be able to determine localization ^40, 48^ or positive staining, but localization was not able to be determined ^50, 51^. Other studies report cytosolic ^52^ or subapical localization ^42^. One study has reported apical membrane localization in cultured choroid plexus epithelial cells ^53^. A common feature of these outwardly directed efflux transporters is a broad substrate specificity and considerable overlap between transporters (see 54). PGP is unusual in that it intercepts lipid-soluble compounds (red symbols, Figure 5) as they pass through the internal leaflet of the plasma membrane and returns them to the extracellular fluid ^55^, whereas BCRP and the MRPs bind their substrates from within the cell cytoplasm. Compounds can be exported from the cell by one or more of these efflux pathways. For example, a lipid-soluble compound that manages to avoid interception by PGP as it passes into the cell may then be metabolized by phase I enzymes (e.g. cytochrome P450 oxidases), conjugated by phase II enzymes (sulfotransferases [SULTs], uridine-diphospho-glucuronosyltransferase [UGT], or glutathione S-transferase [GST]), and exported by BCRP and/or MRP.

There are probably species differences in the level of expression and function of these various efflux transporters, and it is known that their expression changes with age during brain development at both interfaces ^28, 35, 40, 56, 57^. No doubt with further studies other members of this large group of transporters will be found to be functional at one or more of the brain barrier interfaces. ABC transporters that have been identified at different brain barriers are shown in Figure 5, together with an indication of differences in mechanisms of their function.

## Some dogmas and controversies

**Table:** 

**D ogmas A nd C ontroversies I n B rain B arriers B iology** FACT CHECK
***(i)*** *The blood-brain barrier in the embryo and newborn is absent or “leaky”.* Incorrect. Widely believed and stated, usually without experimental basis and in spite of much evidence to the contrary ^2^.
***(ii)*** *Induction of tight junctions in early brain development depends on astrocytes.* Incorrect. Functionally effective tight junctions are present well before the differentiation of astrocytes ^15, 117^.
***(iii)*** *The paracellular pathway (intercellular space) in blood-brain barrier and choroid plexuses is the route for water, small molecules, and ion exchange between blood, brain, and cerebrospinal fluid (CSF).* Most likely not correct. It is based on transepithelial resistance measurements ^118^ with no direct evidence, as water, ions (e.g. Na ^+^), and small lipid-insoluble molecules (e.g. sucrose) cannot yet be visualized with sufficient resolution. Recent methods using visualizable, similar small molecules have shown transfer through epithelial cells of choroid plexus and not via paracellular pathway ^119^. Other limitations of Frömter & Diamond ^118^ are discussed in Ek *et al.* ^119^.
***(iv)*** *Increased penetration of molecules into brain parenchyma in pathological conditions is due to the breakdown of tight junctions at the barrier interfaces.* Probably not correct in many cases. Most studies do not use electron microscopy (EM) required to define state of tight junctions. Evidence is emerging that in e.g. stroke, tight junctions are intact and transfer may be intracellular across cells forming the barrier (e.g. Krueger *et al.* ^120^); some studies have shown ultrastructural changes in both cellular constituents and tight junctions ^121^. In addition, regulation of various transporters may change in some pathologies ^122, 123^.
***(v)*** *Evans blue binds tightly and specifically to plasma albumin and can be used to measure penetration of albumin into brain in barrier dysfunction*. Incorrect. Evans blue binds to multiple proteins in plasma and to cells and tissues in reversible equilibrium ^124^.
***(vi)*** *Lack of lymphatics in brain*. Incorrect. Evidence going back many years suggesting drainage pathway via cervical lymphatics is now supported by evidence from 2-photon microscopical studies ^125^.
***(vii)*** *Brain extracellular space (ECS) volume*. Controversial. Decades-long discrepancy between EM measurements (negligible ECS) and physiological measurements (approx. 15%) has been resolved by cryo-fixation-EM ^126^. Important for interpretation of blood-brain barrier transfer studies.
***(vii)*** *Concentration of markers in the CSF is a measure of blood-brain barrier permeability.* Untrue. Commonly used, especially in human studies, but is misleading because concentration of any molecule in the CSF does not necessarily reflect transfer across the blood-brain barrier ^127^ and is influenced by its transfer through blood/CSF barrier and only indirectly and variably via the blood-brain barrier ^128^; also CSF drainage, uptake into the brain across ependyma ^24^, and possibly lymphatic drainage ^125^ all influence levels in the CSF.
**(viii)** *The brain is a site of immune privilege*. Recent findings of a functional and classical lymphatic system suggest this concept needs further study ^129^.

## Drug targeting to the central nervous system

This has been a major field of endeavor over the past 20–30 years. It has been largely unsuccessful in that few of the proposed methods appear to have been independently replicated, and we are not aware of any neuropharmaceutical drugs that have been identified using these methods. There are several comprehensive reviews of the methods that have been developed ^58– 60^. Here, we provide only a list of these methods ( Table 1), with some observations on their limitations. We deal in more detail with methods designed to allow entry of drugs into the brain by disruption of the blood-brain barrier. This is the only method of drug delivery that has translated to clinical practice, albeit on a limited basis. Newer, more focal methods hold promise of significant advances using this approach.

The following drug delivery approaches have been tried:

(i) *In vitro* blood-brain or blood-CSF barrier models (see reviews in 61– 64)

The hallmarks of success of such systems are generally held to be a high transendothelial resistance (TEER) and limited permeability to barrier integrity markers such as ^14^C-sucrose ^64^. The only *in vivo* TEER values that have been measured are for pial blood vessels ^65^. It is unclear whether these reflect the properties of vessels within the brain. The type of endothelial cells isolated in preparation of the cultures is often not clear ^66^. But perhaps the biggest limitation of these systems is that few attempts have been made to characterize at the molecular level the barrier and transport properties *in vitro* compared to those *in vivo*. This would seem to be particularly important given the propensity for cells to transform in culture. Where attempts have been made, the extent to which the *in vivo* properties are retained is limited ^67^.

(ii) Receptor-mediated and adsorptive-mediated transcytosis (see Table 1)

Lajoie and Shusta ^60^ review a number of more recent developments using alternative targets on cerebral endothelial cells, but it is too soon to tell whether these will be more successful than earlier developed methods.

(iii) Influx transporters

As indicated above, there are numerous influx transporters in brain endothelial cells. In the case of only a few, it has been possible to use these to achieve penetration of a therapeutic compound into the brain. The best known, and one of the earliest to be described, is L-DOPA for the treatment of Parkinson’s disease, e.g. 68. Its introduction transformed the treatment of this condition, but, after prolonged experience, it is clear that it has serious clinical limitations.

(iv) Inhibition of efflux transporters (see above, 69, and Table 1)

The number of ABC transporters that have been shown to be functionally effective at the blood-brain barrier is only a small proportion of the known total of 49. A huge number of drugs and other xenobiotics are excluded from the brain ^70^, which explains the lack of specificity of ABC transporters. Unless some way could be found to limit the effect of the inhibitor to cerebral endothelial cells, and preferably only those in the neurological target area of the brain, this is unlikely to be a viable method of promoting drug entry to the brain.

(v) Modulation of integrity of the blood-brain barrier

Three methods have been tried − osmotic opening, ultrasound, and electrical stimulation ( Table 2).

*Reversible osmotic opening* of the blood-brain barrier was first demonstrated in animals by Rapoport *et al.* ^71^ using a variety of hypertonic electrolyte and non-electrolyte solutions. Brightman *et al.* ^72^ showed that barrier opening to horseradish peroxidase was due to opening of cerebral vessel tight junctions. Since 1979, Neuwelt has pioneered the use of osmotic opening of the blood-brain barrier as a means of delivering chemotherapeutic agents to treat brain tumors ^73^. He has built up an impressive array of animal and patient imaging techniques, which allowed careful evaluation of the use of hypertonic solutions to open the barrier under well-controlled, carefully monitored conditions and to develop methods for mitigating some of the potentially devastating side effects ^74, 75^. Reversible osmotic opening of the blood-brain barrier is the only technique for improving drug delivery to the brain that has successfully translated to the clinic. It is not widely used, probably because it requires repeated hospital admissions and general anesthesia, as well as being associated with increased risk of stroke and epileptic seizures ^76^ and other surgical and neurological problems ^75^.

*Focused ultrasound disruption of the blood-brain barrier* in laboratory animals was first investigated in the 1950s ^77^. In that study and in subsequent ones, it was necessary to perform a craniectomy in order to achieve sufficient ultrasound energy to produce effects in the brain (e.g. 78). A major advance was to combine intravenous injection of gas bubbles, previously developed as a contrast agent for ultrasound imaging, with focused ultrasound ^79^; this reduced the ultrasound power required to disrupt the blood-brain barrier and was shown to be effective through the intact skull in rabbits. Subsequent studies have evaluated the safety of the procedure ^80^, effects of different anesthetic agents ^80^, and feasibility in large animals ^81^. The mechanism of the interaction between the micro-bubbles and the focused ultrasound beam is unclear. Several possibilities are discussed by Burgess and Hynynen ^82^ and by Timbie *et al.* ^83^. The advantages of this approach compared to osmotic disruption of the blood-brain barrier are that (a) it is non-invasive (does not require craniotomy), (b) it can be targeted to a specific lesion, e.g. tumor, or region of neurological disorder such as the basal ganglia in Parkinson’s disease, (c) it is transient, although the estimates of duration barrier opening have varied from 6 to 24 hours in different studies, and (d) under well-defined conditions of ultrasound parameters, there appears to be no evidence of ischemia, apoptosis, or cognitive dysfunction (tested in primates ^82^). Investigations so far have concentrated on the mechanical disruptive effects of the method, but studies are needed to investigate possible effects on cellular transport across cerebral endothelial cells ^84^. Also, it needs to be considered whether the effects might be different in pathological brains.

**Table 1.** Drug targeting to the central nervous system.

Method & Key References	Rationale	Limitations
*In vitro* barrier systems ^61– 64^	Potential for high-throughput screening	*In vitro* transformation of cell properties. Only limited gene expression of *in vivo* characteristics
Receptor- or adsorptive- mediated transcytosis ^60, 108^	Uses known receptors (e.g. Tf, insulin) & cellular mechanisms	Not restricted to brain; only about 15% reaches brain. Limited capacity
***Influx transporters***		
SLC transporters ^109^	Naturally occurring transporters that also transport wide range of drugs	Many of the transporters are ubiquitously expressed, widespread effects likely. Limited transport capacity. Drugs may also be substrates for ABC efflux transporters
***Efflux transporters***		
Inhibition of efflux transporters ^110^	ABC transporters are major reason for drugs not reaching brain	Not restricted to brain, widespread side effects likely from both drug entry into other organs and entry of other xenobiotics that may be present
***Modulation of integrity of the blood-brain barrier***		
See Table 2		
***Bypassing the barriers***		
Convection-enhanced delivery ^111^	Localized delivery to site of pathology	Invasive. Potential damaging effect not yet fully evaluated
Injection into CSF ^112^	Bypasses barriers	Invasive, requires repeated administration or infusion, not targeted to sites of pathology

**Table 2.** Modulation of integrity of blood-brain barrier as method of drug delivery to the brain.

Method and Key References	Level of Preclinical Evaluation	Invasive	In Clinical Use
Reversible osmotic opening ^73– 76^	Substantial	Yes	Yes, in small number of centers
Focused ultrasound + micro-bubbles ^79, 81, 83, 84^	Substantial	No	No
***Electrical stimulation***			
Non-thermal electroporation ^76^	Limited	Yes	No
Pulsed electromagnetic stimulation ^113^			
Sphenopalatine ganglion stimulation ^114, 115^	Limited	Yes	No
Electric field application ^116^	Limited	No	No
Convection-enhanced delivery ^111^	Limited	Yes	No

## What next?

The concentration in the past 25 years on developing *in vitro* systems for testing barrier permeability to drugs and the various drug delivery methods outlined above has been at the expense of fundamental research aimed at better understanding of brain barrier mechanisms. We list here some major questions, the answers to which might aid the development of more effective drug delivery strategies as well as our understanding of the involvement of brain barrier mechanisms in a wide range of neuropsychiatric conditions.

(i) Different approaches to drug development

Given the overwhelming importance of efflux transporters in excluding drugs from the brain, we need better understanding of the molecular nature of their mechanism(s) of action, as this would allow the development of drugs that evade these mechanisms. However, there would still be a need to develop ways of targeting the drugs not just to the cerebral vasculature but also to specific brain regions. This might come from better knowledge of the molecular characteristics of cerebral endothelial and peripheral endothelial cells as well as identifying such differences in different brain regions, as suggested by Pachter’s work ^66, 85^.

(ii) Rapid high-throughput screening of drugs with potential for neurotherapeutic treatments

In the past, most such drugs have been developed *in vitro* but failed as useful agents *in vivo* because of inability to cross the blood-brain barrier ^86^. Two plausible approaches, on which a start has been made, are (a) to use in initial screens organisms that are easily available in large numbers, e.g. flies ^87, 88^ and zebrafish ^89, 90^. These organisms utilize ABC efflux transporters as in mammals, although the morphological sites at which they function are different in invertebrates ^87^ and the actual ABC transporters that are functionally important may be different in different species. Also, (b) we require a better understanding of the ABC transporters in human brain barriers (adult and developing) and their level of function. It should then be possible to devise appropriate cell-based screens using fluorophore-tagged drugs and competitors ^91^.

(iii) What is the risk to the developing brain of drugs administered to pregnant women?

As indicated above, several ABC transporters are expressed at high levels in embryonic brain both in blood vessels and in the choroid plexuses ^28, 35^. However, it is uncertain if expression levels can be equated to functional exclusion of drugs from the brain. If this can be shown, then coupled with similar transporter activity in the placenta, this suggests that the developing brain may be much better protected than is implied by the discredited but still current dogma that the blood-brain barrier in the embryo is unformed or “leaky” (see Text Box). Nevertheless, the loss of the placental protection in prematurely born infants may mean that they are more vulnerable to the ill effects of drugs than their full-term counterparts.

(iv) Involvement of barrier mechanism in brain disorders

The literature on possible involvement of blood-brain barrier mechanisms is too extensive to cover in this review. Recent papers describing different aspects of the pathobiology of brain barrier mechanisms are 92, which is particularly comprehensive, and 69, 93– 95. For decades, the focus has been on dysfunction defined by supposed disruption of the blood-brain barrier often defined with unsuitable markers (see Text Box). Only comparatively recently has attention turned to the possibility that transporter dysfunction may be involved. It is usually unclear whether barrier dysfunction is a cause or a consequence of a particular neurological disorder. This is an area in which further research with modern technology is likely to be fruitful.
